# A resource of data and samples in cognitively unimpaired persons at six levels of Alzheimer's disease risk based on their APOE genotype

**DOI:** 10.1002/alz70856_102172

**Published:** 2025-12-24

**Authors:** Kathryn L. Ghisays, Deborah Brostrom, Robert J. Bauer, Vivek Devadas, Hayley Salata, Christopher T. Brown, Valentina Ghisays, Trisha L Walsh, Winnie Liang, Nicholas J. Ashton, Yonas E. Geda, Matthew J Huentelman, Jeremy J. Pruzin, Yi Su, Richard J. Caselli, Jessica B. Langbaum, Bryan K. Woodruff, Eric M. Reiman

**Affiliations:** ^1^ Banner Alzheimer's Institute, Phoenix, AZ, USA; ^2^ Mayo Clinic Arizona, Scottsdale, AZ, USA; ^3^ Banner Sun Health Research Institute, Sun City, AZ, USA; ^4^ Barrow Neurological Institute, Phoenix, AZ, USA; ^5^ The Translational Genomics Research Institute (TGen‐ an Affiliate of City of Hope), Phoenix, AZ, USA

## Abstract

**Background:**

Apolipoprotein E (APOE), the major genetic risk factor for Alzheimer's disease (AD), has three common alleles (2, 3 and 4), giving rise to genotypes that are associated with six levels of AD risk: (2/2<2/3<3/3<2/4<3/4<4/4). In our new Arizona APOE Cohort Study, we have begun to establish a longitudinal cohort of more than 300 cognitively unimpaired persons (50‐90 year‐old) stratified by APOE genotype and age decile, matched by age and sex, and have an extensive battery of cognitive, clinical, amyloid (NAV4694) and tau (MK6240) PET, MRI, cerebrospinal fluid (CSF), plasma, and DNA. The study is intended to clarify the impact of APOE4 and APOE2 allelic dose on the predisposition to and protection from AD and inform the design of secondary and primary prevention therapies. Here, we describe the study and the resource of data and samples that are now available to researchers.

**Methods:**

Participants were recruited from across the United States using GeneMatch and other sources. Comprehensive clinical assessments, brain images, and lumbar punctures are performed every 2 years, 50mL blood samples are acquired annually, and whole genome sequencing is in progress.

**Results:**

As of January 2025, baseline assessments were completed in 303 participants, 68±9 years of age, including 205 females and 98 males. Sixteen percent are from underrepresented groups. Baseline CSF samples were obtained in 89% of the participants. The complete cohort consists of 17, 63, 41, 65, 66, and 51 persons with the APOE 2/2, 2/3, 3/3, 2/4, 3/4, and 4/4 genotype, respectively. In the first 287 participants with available amyloid PET scans 6% of 2/2, 5% of 2/3, 14% of 3/3, 26% of 2/4, 29% of 3/4, and 52% of 4/4 participants had a positive amyloid PET (≥20 centiloids).

**Conclusions:**

Building up on our decades of experience in APOE research, we launched the new Arizona APOE Cohort which promises to provide a valuable resource of data and samples to advance the study of APOE, its variants and interactions with other risk and protective factors, and the development, treatment, and prevention of AD. Baseline data are now available to interested and eligible research collaborators.

